# Supplementary material for: Ultrafast current imaging by Bayesian inversion
Source: Nat Commun. 2018 Feb 6;9:513. doi: 10.1038/s41467-017-02455-7 (PMC5802759; doi:10.1038/s41467-017-02455-7)
Supplement: Supplementary file 1 — Supplementary Information [file 41467_2017_2455_MOESM1_ESM.pdf]

Supplementary Figure 1

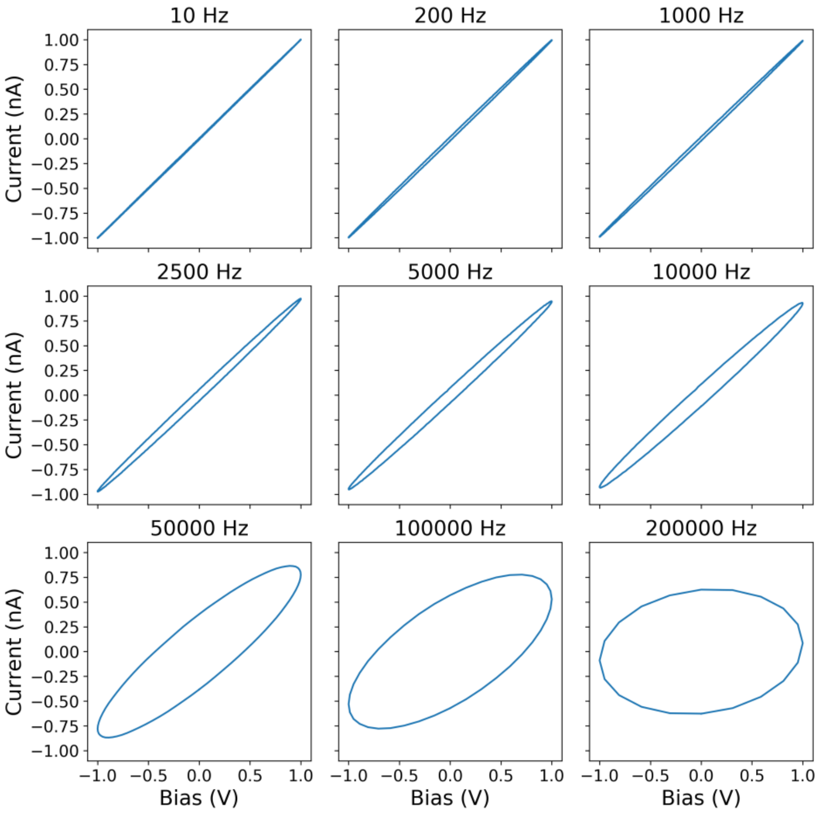

Supplementary Figure 1: Dependence of hysteresis in IV measurements upon excitation frequency for a system consisting of a standard (i.e., constant) resistor

Supplementary Figure 2

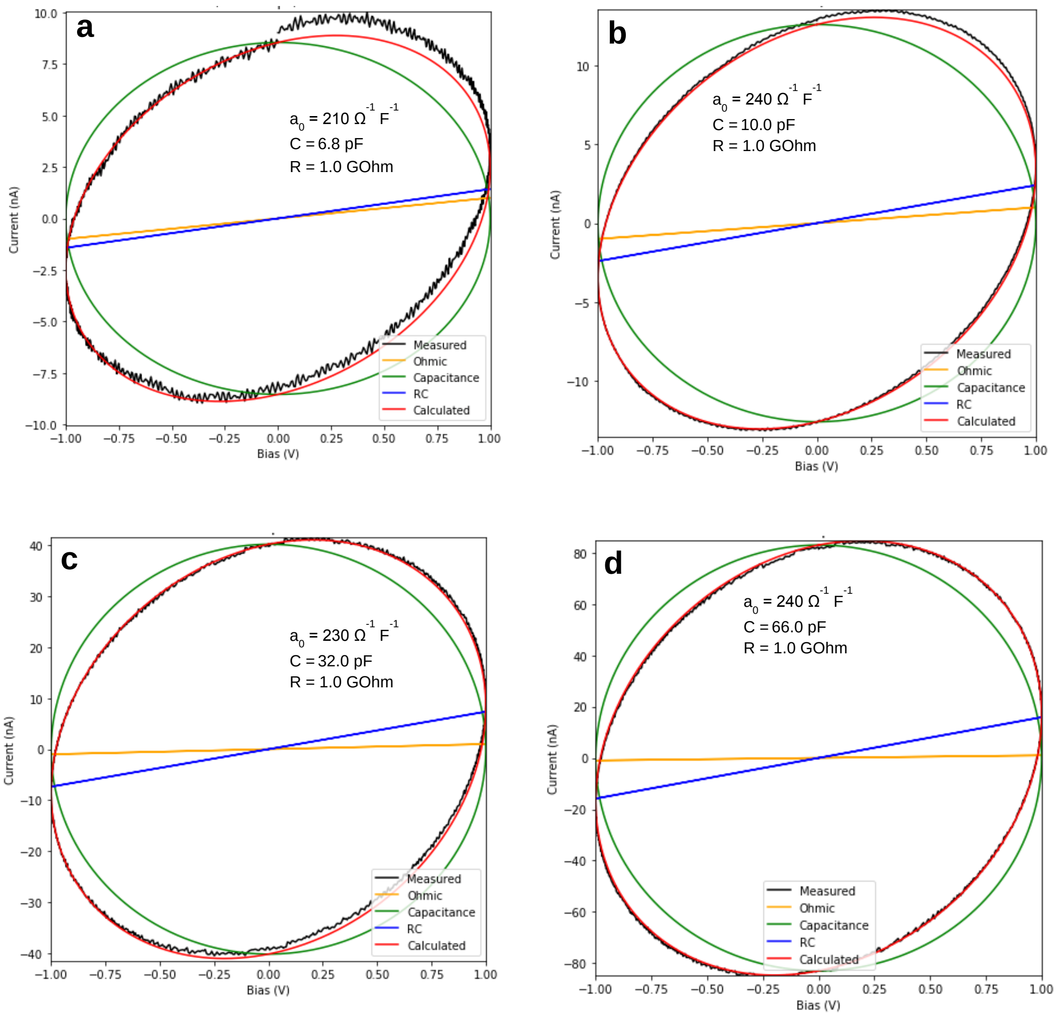

Supplementary Figure 2: Calibration test with simple parallel RC circuit with resistor (R = 1.0 GOhm) and different values of the capacitance *C* for C = (a) 6.8pF, (b) 10.0pF, (c) 32.0 pF and (d) 66.0 pF. The measured current is in black. The pure capacitance contribution to the current (C dV/dt) is plotted in green. The expected Ohmic contribution to the current is plotted in yellow. This leaves an additional Ohmic contribution, which represents the difference, i.e. $I_{measured} - I_{capacitance} - I_{true}$. The value of this contribution appears to be equal to a constant multiplied by the capacitance – a_0C *V. The value of a_0 across this calibration is steady, between 210-240.

Supplementary Figure 3

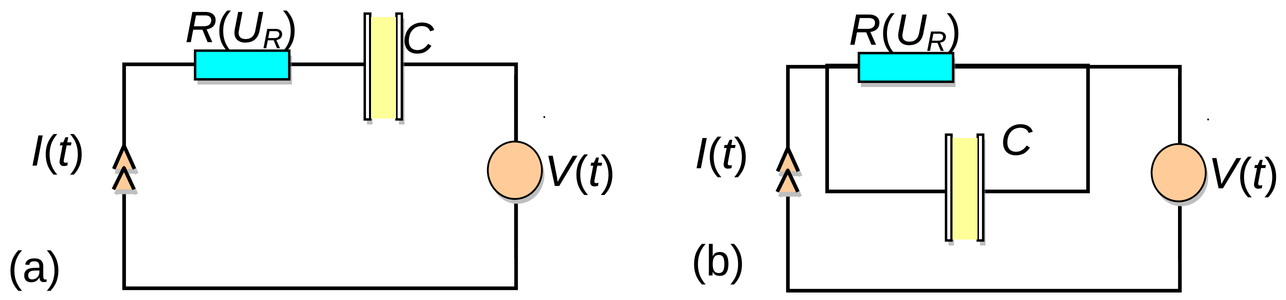

Supplementary Figure 3: Equivalent electric circuit of the considered structure. **(a)** It consists of short-circuited capacitor and resistor in series; **(b)** capacitor and resistor are in parallel.

Supplementary Figure 4

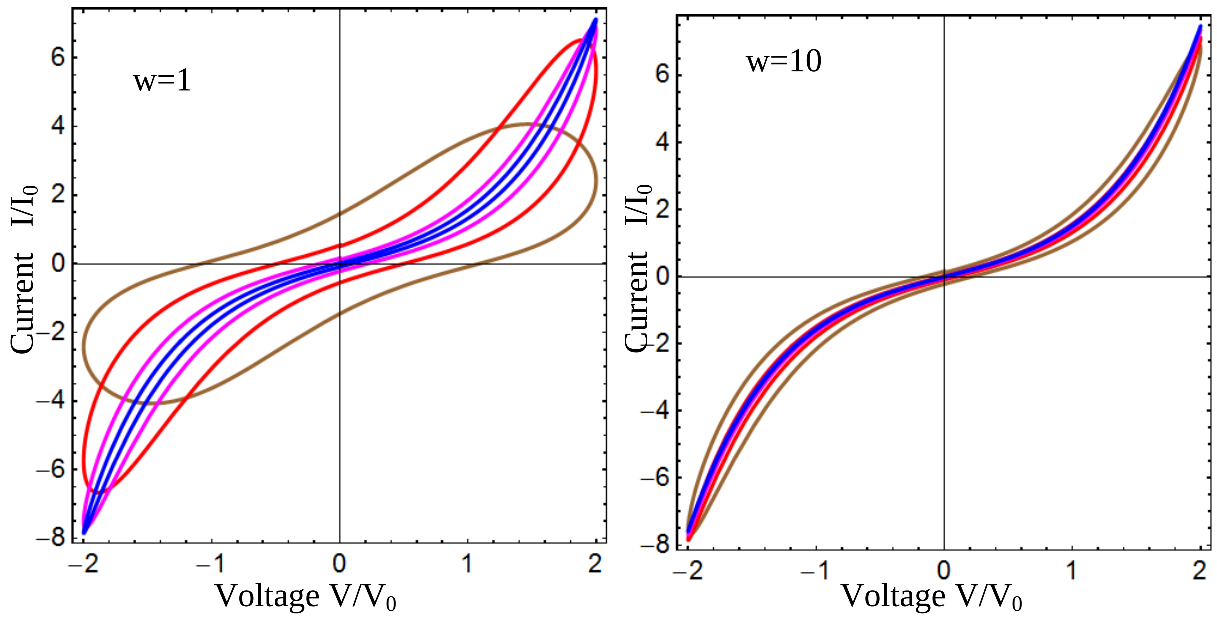

Supplementary Figure 4: *I(V)* plots for different frequency *w* (a, b) and fixed other parameters. Different curves correspond to the different capacity C=3, 10, 30 and 100 (brown, red, purple and blue curves).

Supplementary Figure 5

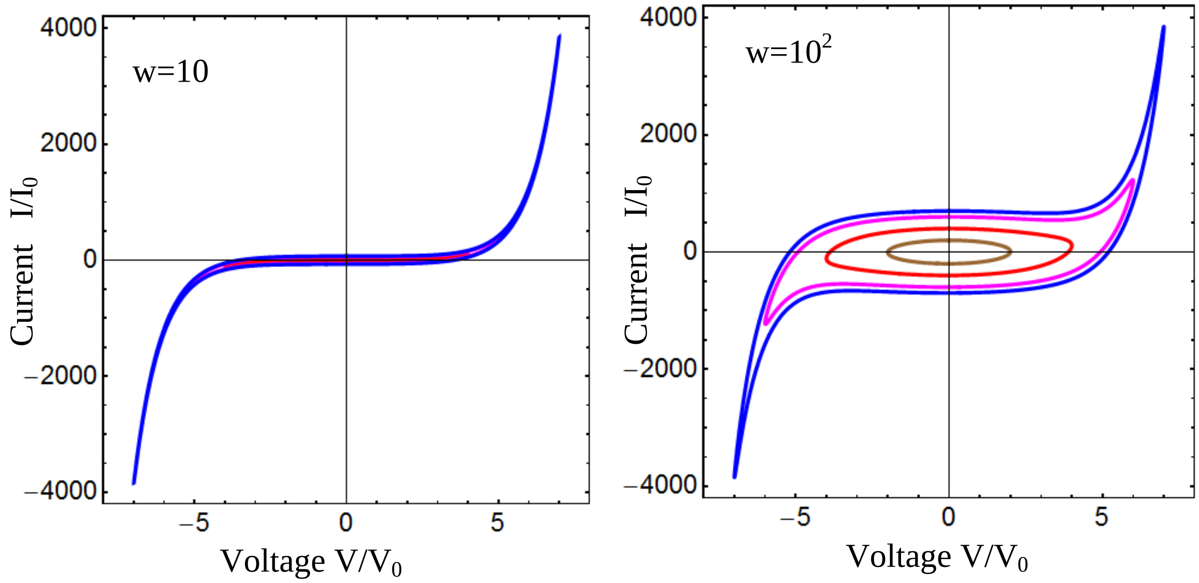

Supplementary Figure 5. *I(V)* plots for different frequency w (in relative units) and fixed other parameters. Different curves correspond to the different amplitude of V/V_0 .

Supplementary Figure 6

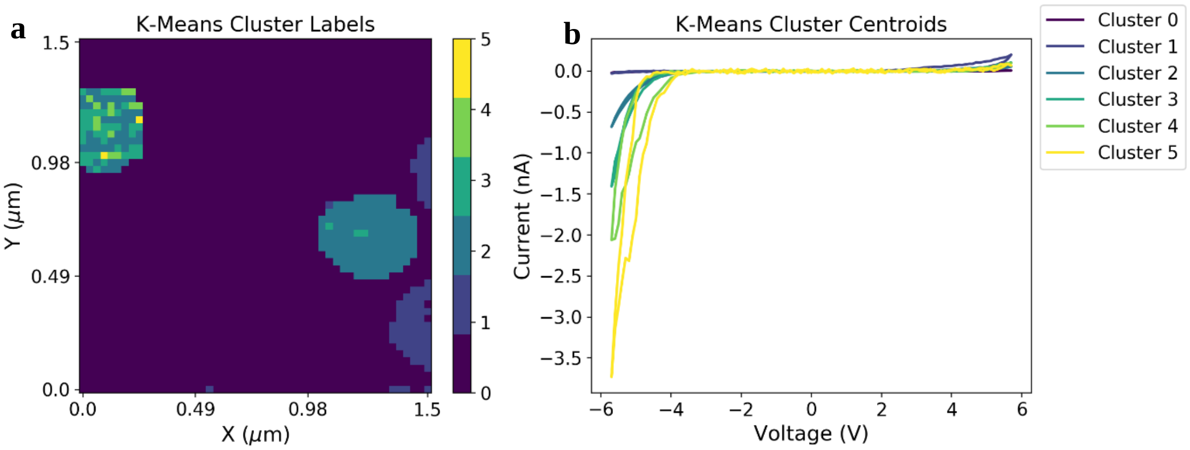

Supplementary Figure 6: To additionally visualize the data from the standard I-V measurement, we present a multivariate statistical analysis approach. Namely, we utilize the k-means clustering algorithm, which attempts to classify a set of *n* observations $(\boldsymbol{I_1}, \boldsymbol{I_2}, \ldots, \boldsymbol{I_n})$ in *d* dimensions into k sets (or clusters) $\boldsymbol{S} = \{S_1, S_2, \ldots, S_k\}$ such that the within-cluster sum of squares is minimized, i.e., solving the problem

$$\arg\min \sum_{j=1}^{k} \sum_{\boldsymbol{I} \in S_j} \left|\left|\boldsymbol{I} - \boldsymbol{\mu}_j\right|\right|^2 \tag{1}$$

where $\boldsymbol{\mu}_j$is the mean of the points in S_j . In our case, *n* defines the number of pixels, and the number of dimensions *d* of each observation $\boldsymbol{I}$ is equal to the number of voltage steps. The number of clusters to partition the data into, ie., *k*, is given by the user. The algorithm is solved using an iterative method until the within cluster sum of squares no longer changes to within a specified tolerance. Here, we performed the K-means clustering algorithm on the standard I-V dataset, using *k*=11. (a, b) show the results, with the spatial variations of the clusters plotted in (a) and the mean response of each cluster plotted in (b). The results indicate that film has very low leakage, although a couple of the capacitors notably display higher levels of conduction. Most of the current exists on the negative voltage side.

Supplementary Figure 7

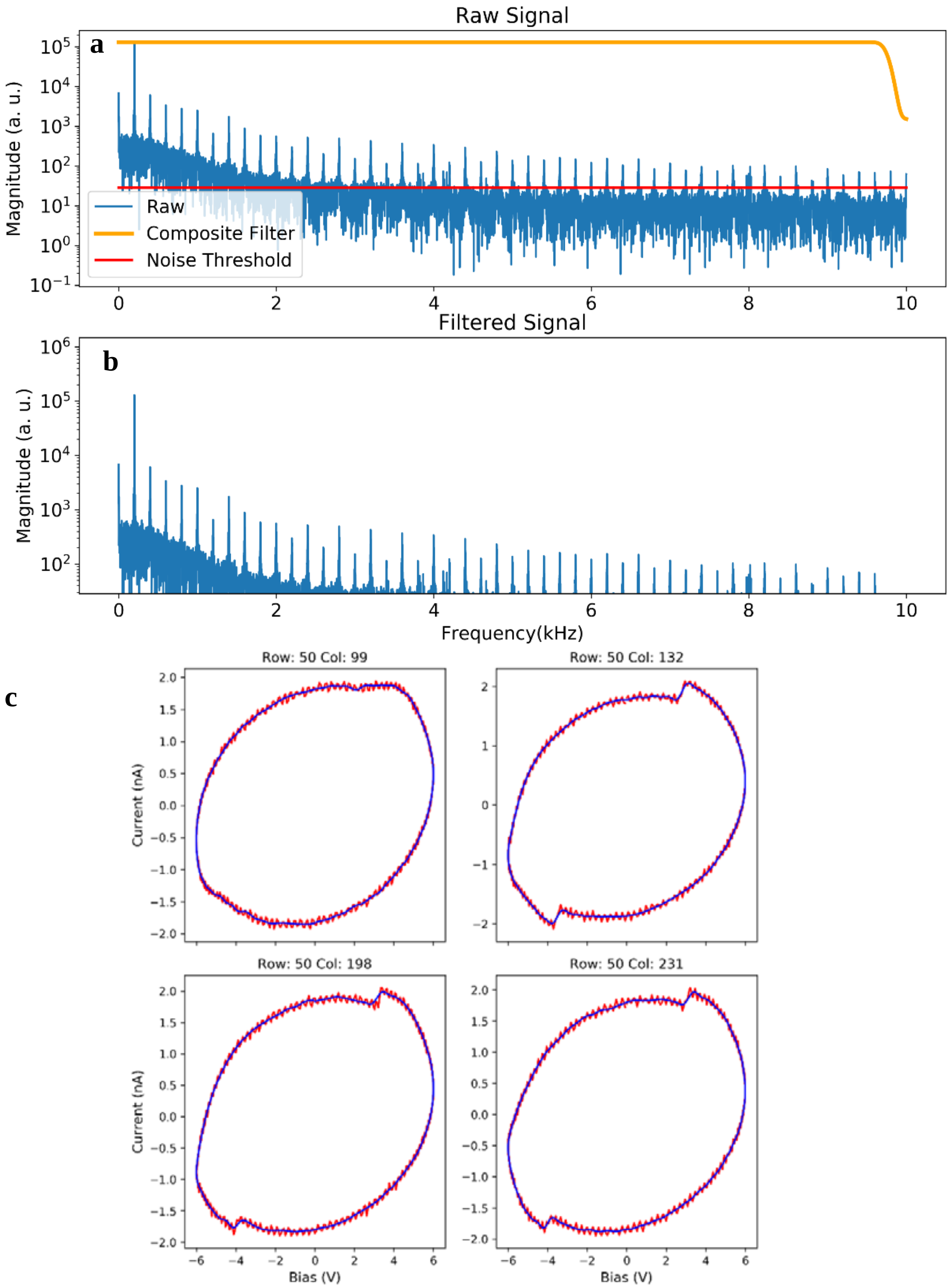

Supplementary Figure 7: Filtering of G-IV Data. (a) Raw current signal from a single line, in the frequency domain. Noise components can be removed by a band-stop filter (none were used here). A noise floor can be set (green line), along with a low-pass filter (orange). (b) Results of filtering the results in (a) with a noise floor and low-pass filter. Shown in (c) are some examples from the same line of pixels before (red) and after (blue) the filtering process.

Supplementary Figure 8

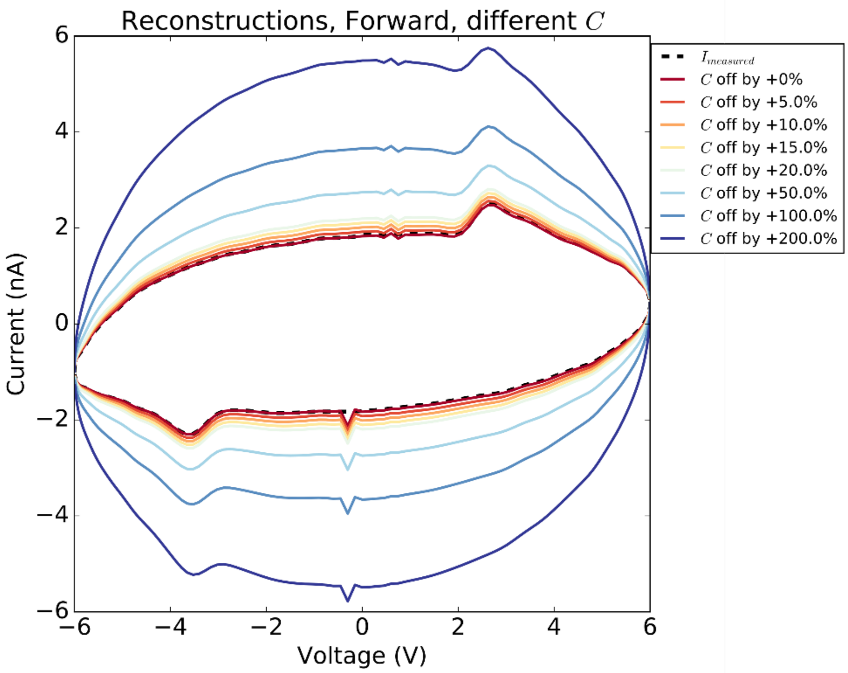

Supplementary Figure 8: Reconstructed current at a single point, based on different values of C.

Supplementary Figure 9

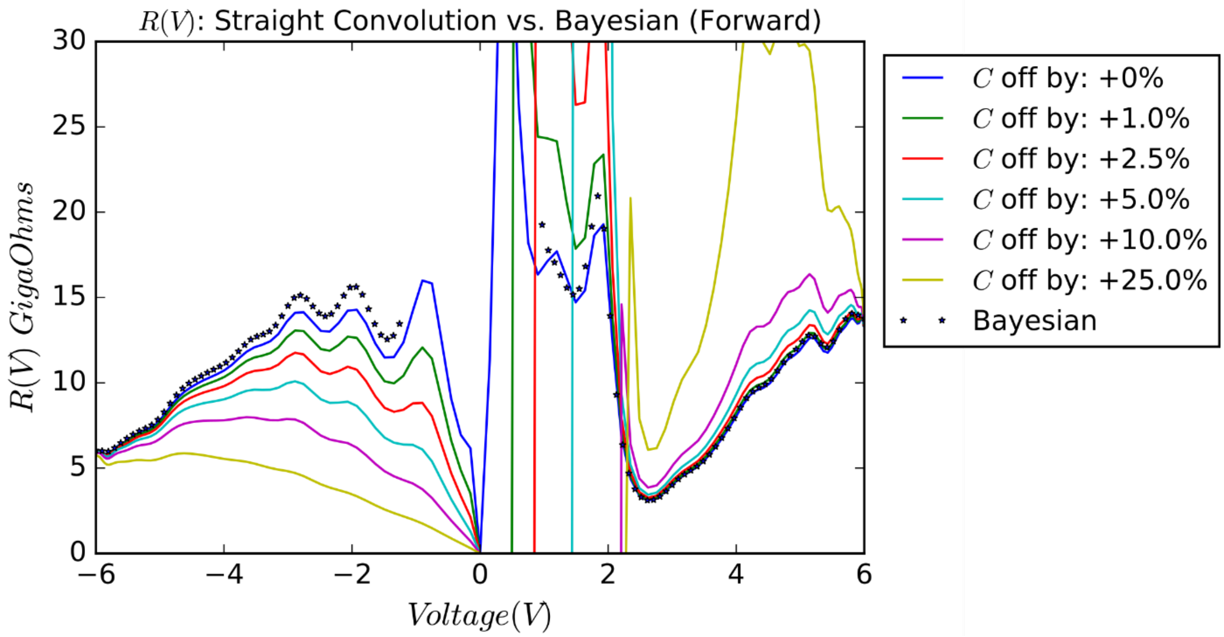

Supplementary Figure 9: Reconstruction of resistance at a single point (forward trace only), based on Bayesian method (diamond), and direct data-driven measurement of *C*. Resistances are off by >GOhm, and uncertainty in the reconstruction is unknown.

Supplementary Figure 10

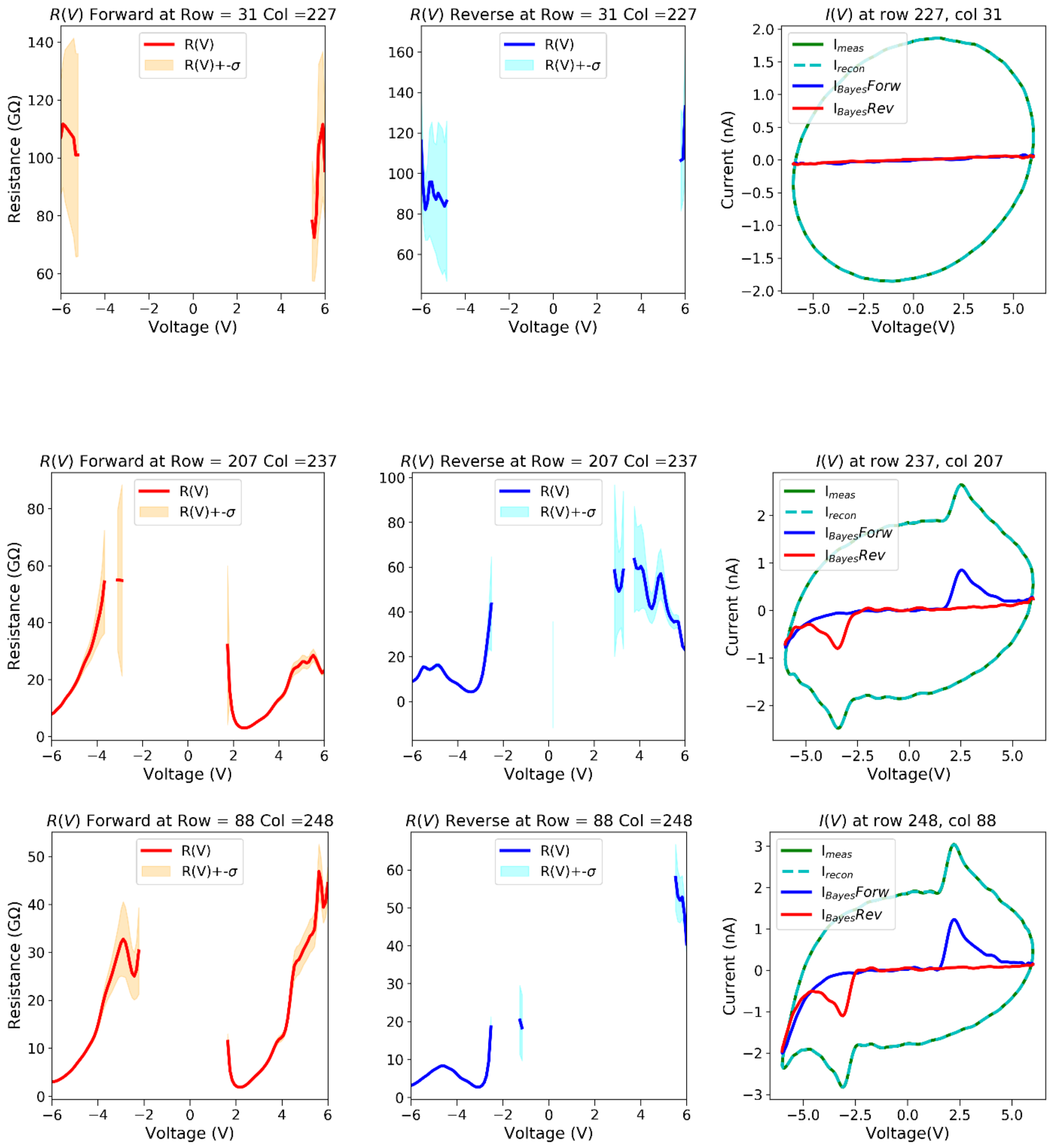

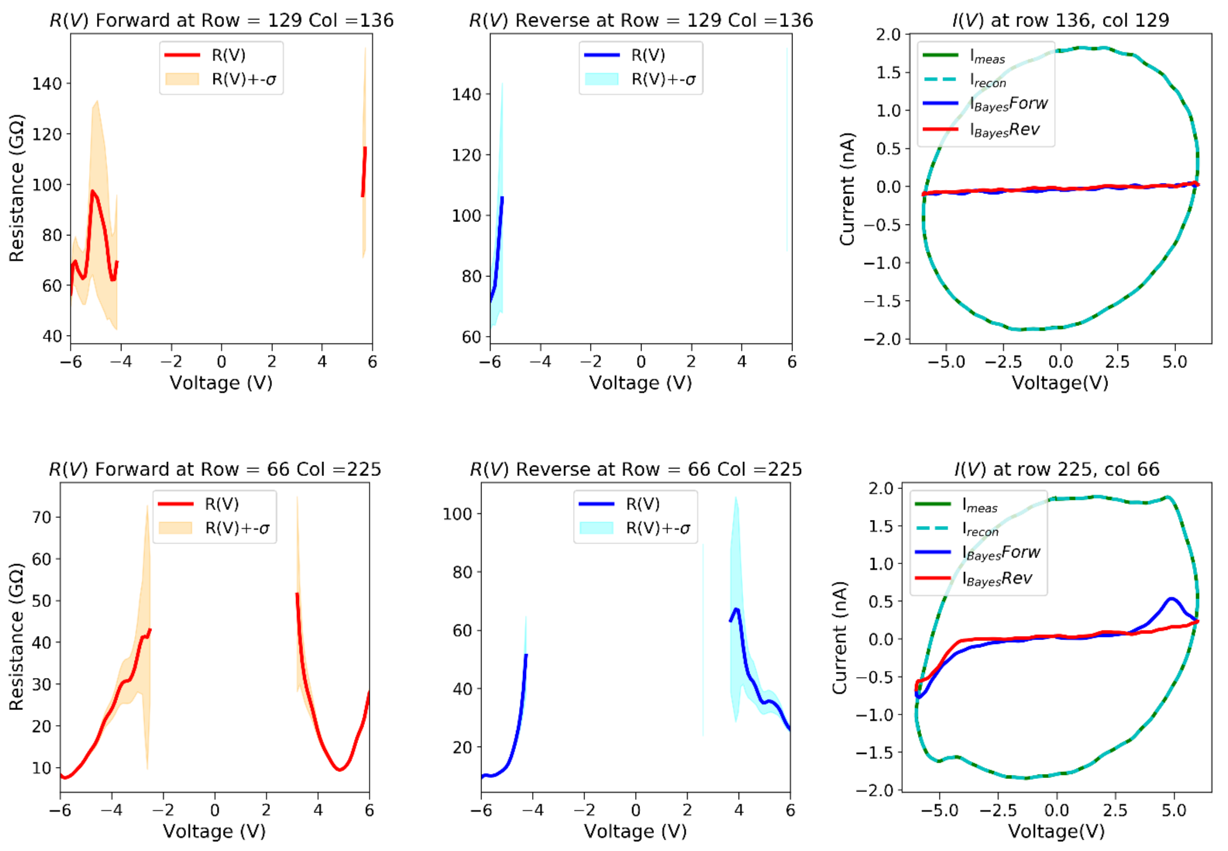

Supplementary Figure 10: The major advantage of Bayesian inference method is the ability to quantify the uncertainty in the reconstruction. Here we plot some further examples of reconstructions. Standard deviations on R(V) inference are shown as filled color areas.

Supplementary Figure 11

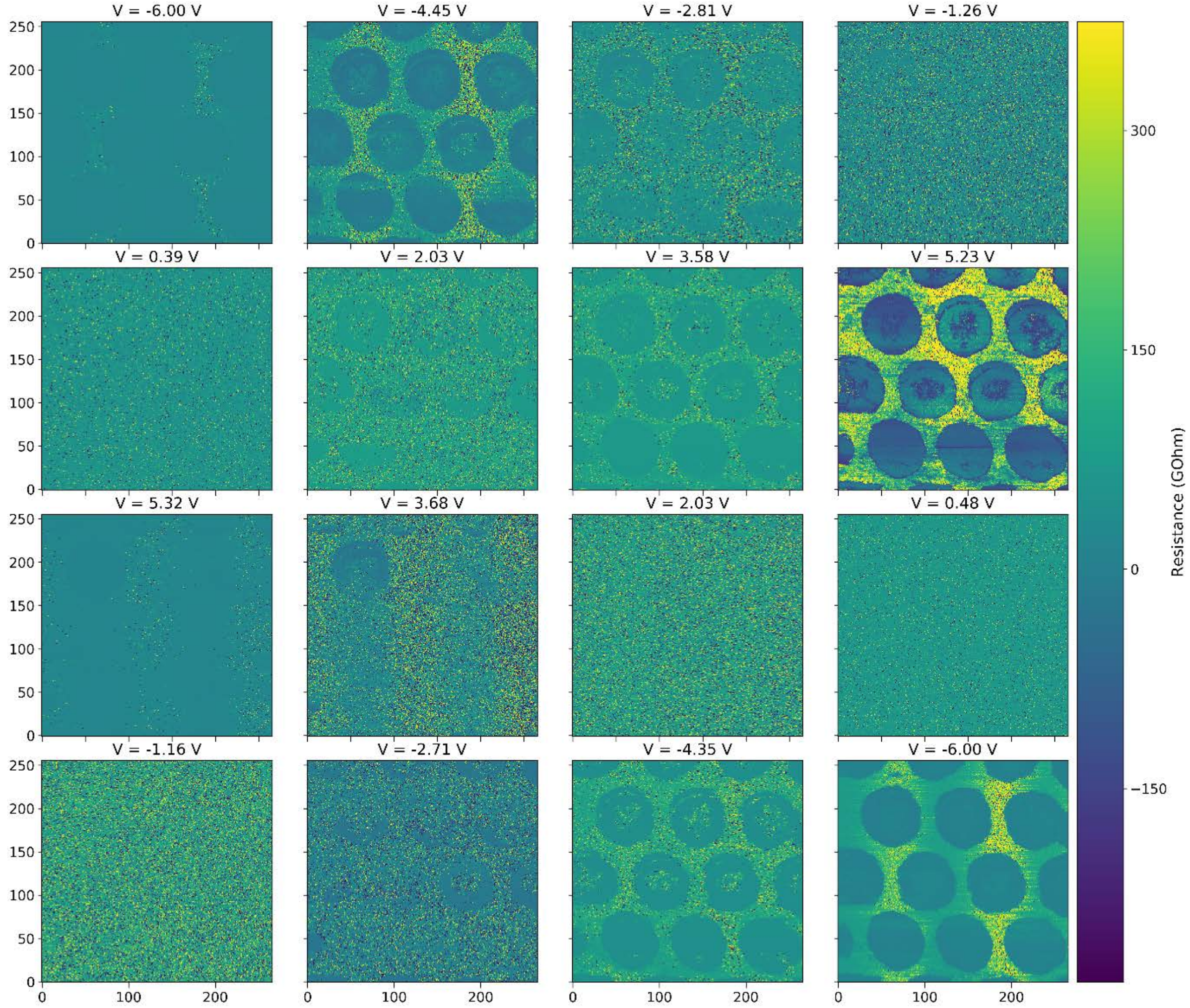

Supplementary Figure 11: Maps of the inferred resistance (*R*) as a function of voltage. The color scale is in GOhms.

Supplementary Figure 12

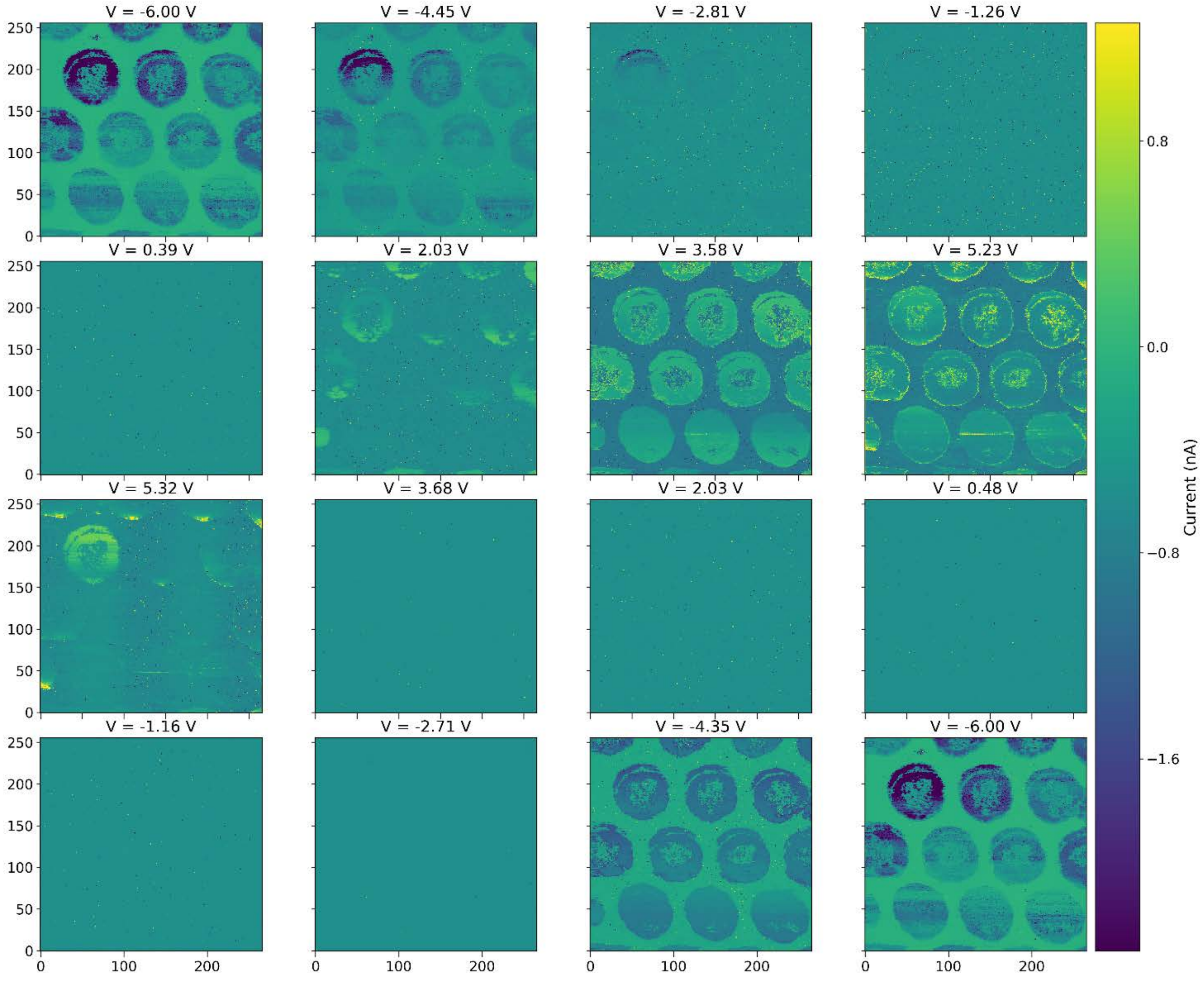

Supplementary Figure 12: Maps of the inferred current (*I*) as a function of voltage. The color scale is in nA.

Supplementary Figure 13

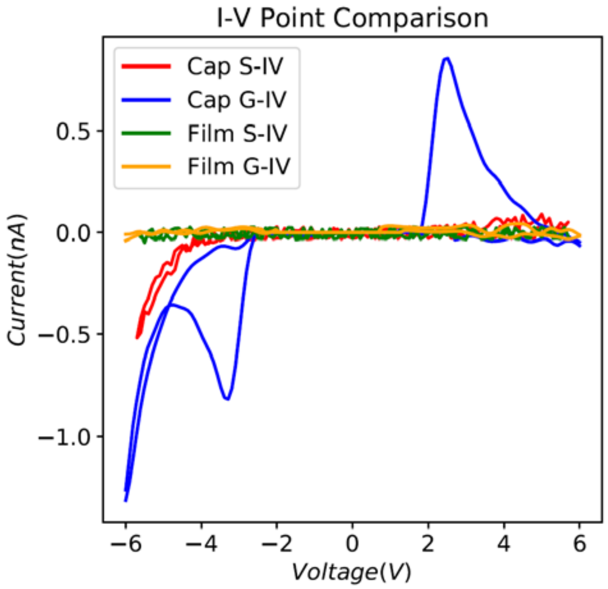

Supplementary Figure 13: To compare the two techniques, we plot the I-V curves on both the capacitor and the bare film, acquired using both the standard method and the G-IV method. The same capacitor was used in this instance for the comparison between the standard and G-IV methods. Red and green lines are from I-V curves acquired using the standard acquisition mode on the cap and film, respectively. The blue and orange lines are from I-V curves taken on the cap and film, acquired using the G-IV mode.

Supplementary Figure 14

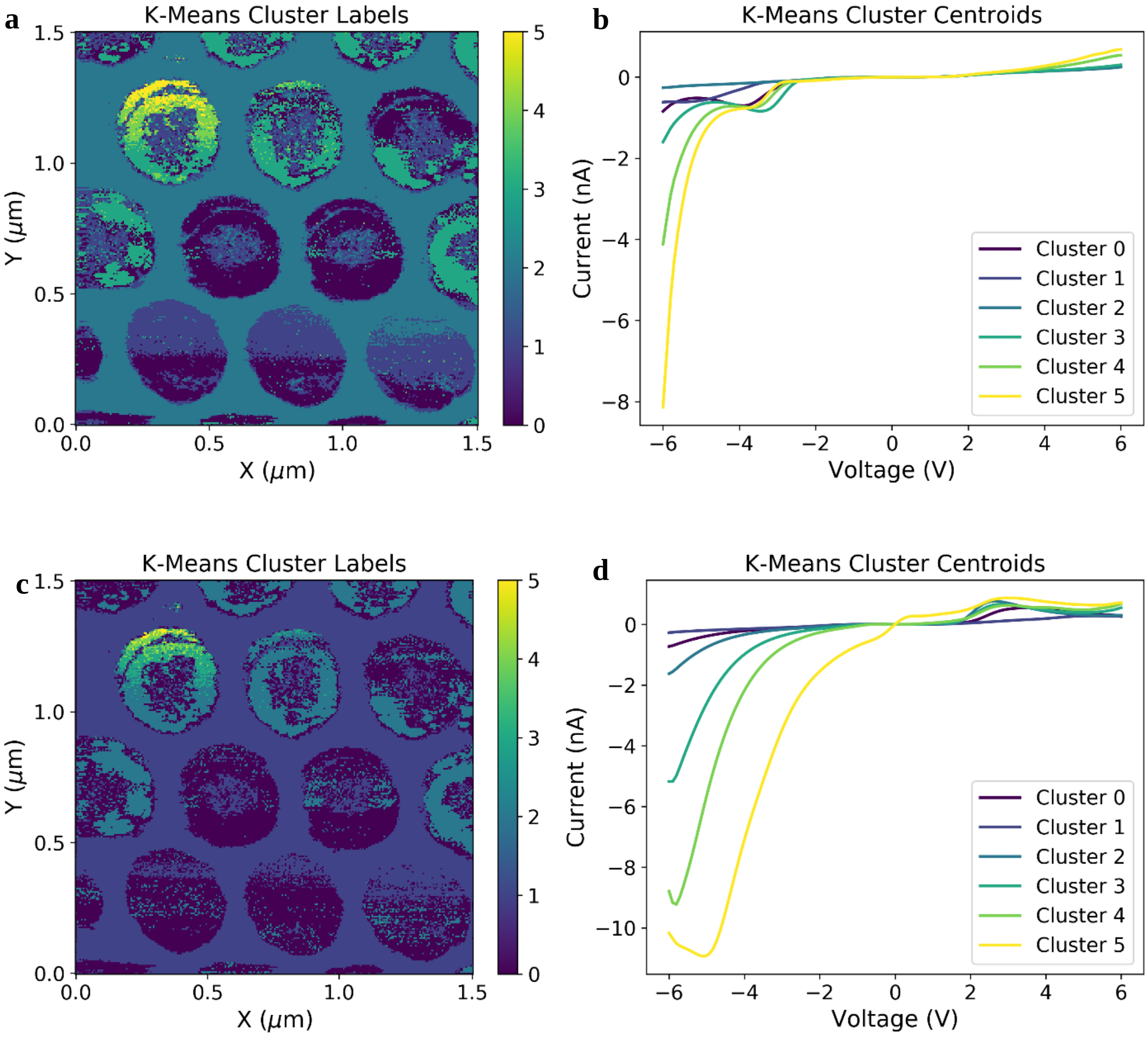

Supplementary Figure 14: To analyze the current further, we proceeded to perform k-means clustering on the G-IV data. The results are shown below for the forward and reverse traces, respectively. (a,b) K-means Results – Forward reconstructed I. (a) Cluster map shown and cluster centers (i.e., mean of each cluster) is shown in (b). The same results for the reverse trace are shown in (c,d).

Supplementary Note 1 – Numerical Modeling

We consider two situations for circuit analysis - one wherein the capacitor is in series with the resistor, and one where the capacitor is parallel to the resistor. In addition to a voltage source, we measure the current *I(t)*, with a constant capacitor with capacitance *C* and non-linear resistor *R*, as shown in Supplementary Figure 3.

We first focus on the series connection case. Consider a periodic voltage $V(t) = U\sin(\omega t)$ is applied to the circuit. The nonlinear equation is:

$$V(t) = I(t)R(U_R(t)) + \frac{Q(t)}{C}, \tag{2}$$

$$R(U_R(t)) = R\left(V(t) - \frac{Q(t)}{C}\right) \approx R(V(t)) - \frac{Q(t)}{C}\left.\frac{dR}{dU}\right|_{U=V(t)}. \tag{3}$$

Here electric charge is $Q(t) = \int_0^t I(t')dt'$, electric current is $I(t) = \frac{dQ}{dt}$, and initial condition $Q(0) = 0$ Voltage at resistor is $U_R(t) = V(t) - \frac{Q(t)}{C}$. Analytical solution of supplementary equation (2,3) are absent. However in the case of large capacitance the series expansion in supplementary equation 3 can be cut at the first term and supplementary equation 2 simplifies to the form:

$$V(t) \approx \frac{dQ}{dt}R(V(t)) + \frac{Q(t)}{C}, \tag{4}$$

The equation can be solved by the constant variation method. Namely $Q(t) = Q_0(t)\exp\left[-\int_0^t \frac{dt'}{R(V(t'))C}\right]$, where the constant $Q_0(t)$ should be varied. The solution that satisfies the boundary condition $Q(0) = 0$ acquires the form:

$$Q(t) = \int_0^t \left(\frac{d\tau V(\tau)}{R(V(\tau))}\exp\left[-\int_\tau^t \frac{dt'}{R(V(t'))C}\right]\right), \tag{5}$$

$$I(t) = \frac{dQ}{dt} = \frac{V(t)}{R(V(t))} - \frac{1}{R(V(t))C}\int_0^t \left(\frac{d\tau V(\tau)}{R(V(\tau))}\exp\left[-\int_\tau^t \frac{dt'}{R(V(t'))C}\right]\right). \tag{6}$$

I(V) plots for different capacity C (in relative units) and fixed other parameters are shown in Supplementary Figure 4 in dimensionless units for voltage V/V_0, current I/I_0 ($I_0 = V_0/R_0$) and frequency $w = \omega/(R_0 C)$. For clarity suppose "two symmetric diode" dependence $R(V(t)) = R_0/(\cosh(V(t)/V_0))$.

Next, we consider the parallel connection case. A periodic voltage $V(t) = U\sin(\omega t)$ is applied to the circuit. The nonlinear equation is:

$$I(t) = \frac{V(t)}{R(V(t))} + C\frac{dV}{dt}, \tag{7}$$

For clarity suppose "two symmetric diode" dependence $R(V(t)) = R_0/(\cosh(V(t)/V_0))$.

Results of *I(V)* characteristics modelling with supplementary equation 7 is shown in Supplementary Figure 5 in dimensionless units for voltage V/V_0, current I/I_0 ($I_0 = V_0/R_0$) and frequency $w = \omega/(R_0 C)$. Different curves correspond to the different amplitude of V/V_0.

Note that supplementary equation 7 allows us to define $R(V(t))$ from measured I(V) characteristics in the evident form:

$$R(V(t)) = \frac{V(t)}{I(t) - C(dV/dt)}, \tag{8}$$

Opening the possibility for the straightforward convolution of the experimental *I(t)* data if *C* is known. Some reasons why this is generally not straightforward are discussed in Supplementary Note 1.

Supplementary Note 2 – Inversion Methods

Here, we wish to discuss some methods of solving the ODE (i.e., equation (2) from the manuscript) to recover the resistance and capacitance values. First, we review the problem: the measurement is an attempt to measure the resistance profile *R(V)* for some measurements *I(t)*, given an applied voltage *V(t)*, i.e.:

$$R(V(t)) = \frac{V(t)}{I(t) - C(dV/dt)} \quad (9)$$

For the sake of simplicity in this example, we have removed the extra term a_0C in this exercise, which can be determined either in an ad-hoc manner or through calibration experiment. Nonetheless so long as the value of a_0 is known, it will not affect the following example of the errors induced by direct inversion.

Several issues arise when one attempts to simply insert the measured value of the current into this equation to yield the resistance. First, the capacitance of the measuring circuit is unknown, and can also be spatially variable (as in the case in the paper, due to the larger contact area afforded by the nanocapacitors as opposed to the smaller contact area afforded by the SPM tip on the bare film). However, it is still possible to progress, if one assumes that the current at "low" voltages arises from purely the capacitive term (i.e., essentially taking the limit that R→infinity for V→0), then one can derive an estimate of the capacitance *C*. But the uncertainty in the measurement of *C* hinders the reconstruction and has a substantial impact. For instance, we have computed the reconstructions of *I* and *R* based on different values of the capacitance. Even a small change in the capacitance term (by ~10%) can result in substantial errors in the inferred current and resistance, as seen in Supplementary Figures 8,9.

Of course, the next challenge is the noisy nature of the measurement. Even given an estimate for the capacitance, direct substitution of *I(t)* and *C* in the equation above would yield very noisy reconstructions of *R*, because the current term lies in the denominator. But perhaps the largest problem with such methods is the inability to quantitatively ascertain the regions wherein the reconstruction can be trusted, and where it should be discarded. By using the Bayesian approach, the above limitations are removed, and the estimator provides a measure of the uncertainty, allowing removal of regions where the data is insufficient to reconstruct the resistance profile.

From the literature, some researchers have previously utilized an AC excitation in the measurement of IV curves, although none used Bayesian inference to infer the capacitance and resistance. For instance, Komoto *et al.*[1] acquired hundreds of I-V curves of a single metal/molecule junction using an STM tip. The paper does not detail the precise method of removing the capacitive contribution, but it appears to be based on a separate reference measurement of *C*. Meyer et al.[2] utilized AC excitations and frequency sweeps to distinguish between the ferroelectric, leakage and capacitive contributions to the measured current, and applied it to observe switching in capacitors on an 180 nm PZT film, with measurements of the current at up to 200 Hz. Here, their approach was to utilize excitations at two distinct frequencies to deconvolute the response. It should be noted this was a macroscopic measurement, which did not use AFM.

For investigations of switching currents with an AFM tip, Martin *et al.*[3] developed a scheme to utilize an AFM setup and subtract the capacitance contribution to the measurement via a reference measurement, enabling detection of displacement currents in PZT films, and allowing for an estimate of the effective tip-surface contact area.

Other methods to remove a parasitic influence of the measuring system capacitance have also been discussed in the literature.[4, 5] However, none of these methods is universal, and several require separate calibration experiments, in contrast to the Bayesian inference method described, which does not require any additional circuitry, handles spatial variation of the capacitance, and also provides a measure of the uncertainty in the reconstruction.

Supplementary Method 1

Before G-IV was applied to image the sample, we characterized the instrumentation by measuring the current response to bias waveforms applied across a standard 1 GΩ carbon-film resistor. The 10 Hz (low-pass) filter on the current-amplifier was turned off to maximize the bandwidth. As expected, the DAQ sampling rate had no effect on the measured response. Furthermore, the amplitude of the bias waveform did not affect the hysteresis in the current measurement. We also observed that increasing the gain in the current-amplifier increased the signal-to-noise ratio at the cost of hysteresis in the signal. This is expected since the bandwidth of the current amplifier decreases with increasing gain. Supplementary Figure 1 shows that as the bias frequency was increased from 10 Hz to 200 kHz, the hysteresis in the measured signal increased dramatically.

We determined the appropriate circuit model using further calibration experiments by using idealized circuits with known resistances and capacitances. This procedure informed us that the inversion procedure can be improved via addition of a small a_0C term, i.e. instead of I = (V/R) + C dV/dt, we found better agreement with

$$I = V\left(\frac{1}{R} + a_0 C\right) + C\frac{dV}{dt} \qquad (10)$$

This value of a_0 varies depending on cabling, but is typically in the 50-300 F^{-1} $Ohms^{-1}$ range. The calibration experiment results are shown in Supplementary Figure 2. Thus, we utilized the parallel RC circuit model with a small correction from an additional a_oCV term.

Supplementary Method 2

Consider the case that voltage is known, and given by $V = V_0\sin(\omega t)$. These are related to the current I, resistance *R(V)*, and capacitance *C*, via the following ODE, where $R: [-V_0, V_0] \to \mathbb{R}_+$:

$$I = V(R^{-1}(V) + a_0 C) + C\frac{dV}{dt} \tag{11}$$

Using the definition above for *V* this can be re-written pointwise as follows

$$I(t) = V_0 \sin(\omega t)\, R^{-1}(V_0 \sin(\omega t)) + CV_0(\omega \cos(\omega t) + a_0 \sin(\omega t)) \tag{12}$$

for each $t \in \mathbb{R}_+, \mathbb{R}_+ = \{t \in \mathbb{R}, t \geq 0\}$. Defining $G(R,C) = \{I(t_j; R, C)\}_{j=1}^{N}$, noisy observations of the current of the following form will be made

$$y = G(R,C) + \eta, \qquad \eta \sim N(b,\Gamma), \quad \eta \perp (R,C) \tag{13}$$

where $N(b,\Gamma)$ denotes a Gaussian random variable with mean b and covariance Γ. The posterior distribution on (R,C)|y is then known pointwise up to a normalizing constant

$$P(R,C|y) = \frac{1}{Z} \exp\left(-\frac{1}{2} \,|\, \Gamma^{-1/2}\,(y - b - G(R,C))|\ \hat{}2\right). \tag{14}$$

After *R* has been approximated by a finite number of parameters, many general Monte Carlo methods exist for probing such posterior distributions, such as Markov chain Monte Carlo methods[6]. However, for our purposes here an alternative statistical model will be preferred, leading to a much faster reconstruction after some post-processing.
Changing variables to $S = R^{-1}$, supplementary equation 12 can be written as

$$I(t) = V_0 \sin(\omega t)\, S(V_0 \sin(\omega t)) + CV_0(\omega \cos(\omega t) + a_0 \sin(\omega t)) \tag{15}$$

for each $t \in \mathbb{R}$. Assume that S can be well-approximated by an expansion of basis functions so that $S(x) \approx \sum_{i=1}^{M} s_i \phi_i(x)$, and denote $\bar{s} = [s_1, \dots, s_M]^T$ and $s = [\bar{s}^T, C]^T$. For a given time series $TS = [0, \tau, 2\tau, \dots, (N-1)\tau]^T$, we define $A \in \mathbb{R}^{N \times M}$ to be the matrix such that

$$\bar{A}_{nk} = V_0 \sin(\omega\tau(n-1))\, \phi_k(V_0 \sin(\omega\tau(n-1)) \tag{16}$$

Define dV as the vector with entries $dV_n = V_0 \omega \cos(\omega\tau(n-1))$. For convenience, we define the matrix A by $A_{:,1:M} = \bar{A}$ and $A_{:,M+1} = dV + a_0 V$. Then supplementary equation 15 can be evaluated at T, and can be written as

$$I(s) = \bar{A}\bar{s} + C(dV + a_0 V)\,. \tag{17}$$

Now, consider the inverse problem of reconstructing S given noisy measurements of

$$y = A\bar{s} + CdV + \eta, \qquad \eta \sim N(b,\Gamma), \quad \eta \perp s \tag{18}$$

where $N(b,\Gamma)$denotes a Gaussian random variable with mean b and covariance Γ (and it is understood that the actual data y from the experiment will come from a true S which may not be exactly represented by the M-term expansion). It will be assumed that the bias b and covariance Γ corrupting the observations are known, and a_0 is also known.

Endow s with a Gaussian prior $s \sim N(0, \mathcal{C})$. Then it is known that the posterior distribution of $s|y$ is given by $N(m, \Sigma)$, where[7]

$$m = \Sigma \mathrm{A}^{\mathrm{T}} \Gamma^{-1} (y - b - I_0) \tag{19}$$

$$\Sigma = (\mathrm{A}^{\mathrm{T}} \Gamma^{-1} A + \mathcal{C}^{-1})^{-1} \tag{20}$$

This can be derived by observing that the unnormalized posterior is the exponential of the following quadratic function

$$\frac{1}{2}\left|\Gamma^{-\frac{1}{2}}(y - b - I_0)\right|^2 + \frac{1}{2}\left|C^{-\frac{1}{2}}s\right|^2, \tag{21}$$

and completing the square for *m* and Σ to write it as follows

$$\frac{1}{2}\left|\Gamma^{-\frac{1}{2}}(y - b - I_0)\right|^2 + \frac{1}{2}\left|C^{-\frac{1}{2}}s\right|^2, \tag{22}$$

Many expectations can be computed analytically for Gaussian distributions. For general $\varphi: L^2([-V_0, V_0]) \to \mathbb{R}$, the posterior expectations with respect to *S* are computed as follows

$$\mathbb{E}(\varphi(S)|y) = \int_{\mathbb{R}^M} \varphi\,(\langle\Psi|\bar{s}\rangle)\mathbb{P}(d\bar{s}|y), \tag{23}$$

where $\Psi(\mathrm{x}) \coloneqq [\phi_1(x), \ldots, \phi_{\mathrm{M}}(x)]^T$, i.e. $S(x) = \langle\Psi(\mathrm{x}), \bar{s}\rangle$. This can be approximated easily using Monte Carlo

$$\mathbb{E}(\varphi(R)|y) \approx \frac{1}{N_S}\sum_{i=1}^{N_S} \varphi\big(\langle\Psi|\bar{s}^{(i)}\rangle\big), s^{(i)} \sim N(m, \Sigma). \tag{24}$$

Clearly this reduction to (*M*+1)(*M*+2)/2 parameters vastly simplifies the general problem of probing the posterior distribution of $R|y$, even in the case R is parameterized with M parameters. However, the price we pay is that $\mathbb{E}(R) = \mathbb{E}\left(\frac{1}{\bar{s}}\right) = \infty$, i.e. the posterior distribution on R is fat-tailed and intractable with infinite moments. We can however compute $1/\mathbb{E}(\langle\Psi|\bar{s}\rangle)$, assuming that the denominators are positive. We can also impose positivity by replacing the density of $(\bar{s})$, $\rho_{\bar{s}}$, with $\tilde{\rho}_{\bar{s}} = \rho_{\bar{s}}\mathbf{1}\{\bar{s} > \varepsilon\}$. This density can be sampled form by simply discarding samples for which $\bar{s} < \varepsilon$. Using this strategy and computing $\mathbb{E}(R)$ and σ_R, as well as simply using $1/\mathbb{E}(\bar{s})$ both provide reasonable reconstructions at a vast reduction in cost in comparison to fully Bayesian direct inversion for *R*. Furthermore, for large values of *R* and small values of *V*, the posterior distribution can become quite complicated and the Monte-Carlo Markov Chain is susceptible to getting stuck for arbitrary starting points. Below, we describe how to construct a reasonable estimate from *S*.

Note that supplementary equation 18 provides a valid and reasonable statistical model, despite the fact that *R* = 1/*S* is undefined, as described above. We can however compute the estimators $\hat{R} := 1/\mathbb{E}(S|y)$ and $\hat{C} = \mathbb{E}(C|y)$, and define error bars by $\hat{R}_{\pm} \coloneqq 1/(\langle\Psi|\mathbb{E}(\bar{s}) \pm \sigma_{\bar{s}}\rangle)$ and $\hat{C}_{\pm} = \mathbb{E}(C|y) \pm \sigma_C$, where $(\sigma_{\bar{s}}, \sigma_C)$ are the posterior standard deviations of $(\bar{s}, C)$, assuming that the denominators are always positive.

Notice that given this estimator, we can define the reconstructed current estimator $\hat{I} := I(\hat{R},\hat{C}) = \mathbb{E}(I|y)$, where the second equality follows from the valid statistical model in supplementary equation 18. Furthermore, the posterior variance of the current is given by

$$\mathbb{E}\left[\left(I - \mathbb{E}(I|y)\right)^2 |y\right] = \left(I(\hat{R}_+, \hat{C}_+) - I(\hat{R},\hat{C})\right)^2 = V^2\sigma_{\bar{s}}^2. \quad (25)$$

If the denominators of the estimators $\hat{R}, \hat{R}_\pm$are not positive, then one must impose a bias in the reconstruction, so that the positivity constraint is preserved, e.g. $\hat{R} = 1/\langle\Psi|\max\{\mathbb{E}(\bar{s}),\varepsilon\}\rangle$, for some fixed $\varepsilon > 0$. More elegantly, alternatives were explored, for exampling imposing the functional form $R = \exp(u)$, and using fully nonlinear Bayesian Markov-Chain Monte-Carlo for sampling from $p(u|y)$. However, for the examples considered, the improvement in the reconstruction was not worth the considerable extra effort in computing posterior estimators. In particular, for small values of V and large values of R the information content of the data is limited and the reconstruction is not impressive in either case. These are also the cases where the *ad-hoc* implementation of the positivity needs to be imposed. For other cases where the information content is high, the methods agree reasonably well, both with each other and with the observed data. Also, the physically constrained fully Bayesian approach is very sensitive to the parameters defining the corresponding statistical model, and so these parameters should be considered hyper-parameters and tuned, either within a jointly Bayesian framework or with an offline maximum likelihood approach. This is a direction for future research, which is expected to pay off in case much higher accuracy is required even where limited information is available.
The basis functions ϕ_i are defined as follows, for some mesh $\{x_i\}_{i=1}^M$

$$\phi_j(x) = \begin{cases} \frac{x - x_{j-1}}{x_j - x_{j-1}}, x \in [x_{j-1}, x_j] \\ 1 - \frac{x - x_j}{x_{j+1} - x_j}, x \in [x_j, x_{j+1}] \\ 0, \quad \text{else}\,. \end{cases} \quad (26)$$

For this choice of basis the function the expansion $\langle\Psi(\mathrm{x}), a\rangle, a \in \mathbb{R}^M$, is a piecewise linear interpolant through the points $\{(x_j, a_j)\}$.

Supplementary References

1. Komoto Y, Fujii S, Nakamura H, Tada T, Nishino T, Kiguchi M. Resolving metal-molecule interfaces at single-molecule junctions. *Sci Rep* **6**, 26606 (2016).

2. Meyer R, Waser R, Prume K, Schmitz T, Tiedke S. Dynamic leakage current compensation in ferroelectric thin-film capacitor structures. *Appl Phys Lett* **86**, 142907 (2005).

3. A new technique based on current measurement for nanoscale ferroelectricity assessment: Nano-positive up negative down. *Rev Sci Instrum* **88**, 023901 (2017).

4. Direct hysteresis measurements of single nanosized ferroelectric capacitors contacted with an atomic force microscope. *Appl Phys Lett* **79**, 3678-3680 (2001).

5. Estevez I, Chrétien P, Schneegans O, Houzé F. Specific methodology for capacitance imaging by atomic force microscopy: A breakthrough towards an elimination of parasitic effects. *Appl Phys Lett* **104**, 083108 (2014).

6. Robert C, Casella G. A short history of Markov Chain Monte Carlo: subjective recollections from incomplete data. *Statistical Science*, 102-115 (2011).

7. Law K, Stuart A, Zygalakis K. *Data Assimilation*. Springer (2015).
