# Supplementary material for: Ultrafast current imaging by Bayesian inversion
Source: Nat Commun. 2018 Feb 6;9:513. doi: 10.1038/s41467-017-02455-7 (PMC5802759; doi:10.1038/s41467-017-02455-7)
Supplement: Supplementary file 3 — Description of Additional Supplementary Files [file 41467_2017_2455_MOESM3_ESM.pdf]

Description of Additional Supplementary Files

File Name: Supplementary Movie 1
Description: Current maps from the standard IV plotted alongside the GIV maps, at each voltage, for the forward direction sweep.

File Name: Supplementary Movie 2
Description: Current maps from the standard IV plotted alongside the GIV maps, at each voltage, for the reverse direction sweep.
